# The value of connected health information: perceptions of electronic health record users in Canada

**DOI:** 10.1186/s12911-016-0330-3

**Published:** 2016-07-16

**Authors:** Sukirtha Tharmalingam, Simon Hagens, Jennifer Zelmer

**Affiliations:** Canada Health Infoway-Inforoute Santé du Canada, 150 King St. W., Ste 1300, Toronto, M5H 1 J9 ON Canada

**Keywords:** Electronic health records, Health information exchange, Evaluation, Benefits, Interoperability

## Abstract

**Background:**

As health care becomes more complex, it becomes more important for clinicians and patients to share information. Electronic health information exchange can help address this need. To this end, all provinces and territories (PTs) in Canada have created interoperable electronic health records (iEHRs). These secure systems offer authorized users an integrated view of a person’s healthcare history across the continuum of care. They include information such as lab results, medications, diagnostic images, clinical reports and immunization profiles. This study explores user experiences and perceived outcomes of iEHR use.

**Methods:**

Surveys conducted between 2006 and 2014 asked iEHR users in six Canadian PTs about system, information and service quality; iEHR use and user satisfaction; and net quality and productivity benefits. The surveys had a core set of questions that used Likert-type scales. Results were synthesized across surveys for each evaluative dimension. Consensus among researchers and subject matter experts on whether to classify the outcomes as positive, mixed/neutral, or negative was established using a modified Delphi technique.

**Results:**

A total of 2316 iEHR users responded to the six surveys. Information quality was the most studied area. Results varied across PTs, but positive outcomes were more common than mixed/neutral or negative outcomes by a 19:1:1 ratio across this dimension. The next most frequently studied aspects were user satisfaction, the impact of iEHR use on quality of care, and the impact on productivity. In all three areas, there were more positive than mixed/neutral or /negative results (ratios of 13:1:1, 14:3:1, and 15:2:1respectively).

**Conclusions:**

Overall, users of iEHRs that provide secure access to patient information collated from across the health system tend to report positive outcomes, including quality of care and productivity. This study is an important first step in understanding user perspectives on iEHRs and health information exchange more broadly.

## Background

The complexity of health care is growing. On average, we see more health care providers, take more medications, and access a broader range of health services than in the past. This means that it is increasingly important that health care providers who care for the same patient share information. As a result, electronic health information exchange is becoming more common around the world with the aim of supporting better access to care, quality, productivity, and patient experiences.

In Canada, the federal and provincial/territorial (‘PT’) governments began investments in the creation of interoperable electronic health records (iEHRs) more than a decade ago. These secure and private systems available to authorized users offer an integrated view of an individual’s health and health care history. Information in iEHRs can come from PT databases, as well as point of care electronic systems in primary care, public health, hospitals, and elsewhere. Lab results, medications, diagnostic images, clinical reports, and immunization profiles are stored and shared by iEHRs. These iEHRs are closely related to what is often referred to as “health information exchange” (HIE) in other countries [1]. More specifically in the United States, HIE refers to the reliable and interoperable electronic sharing of patient’s vital medical information securely among a variety of health care stakeholders (clinicians, laboratories, hospital, pharmacy, health plans, payers and patients) [2, 3].

Canada Health Infoway (Infoway) measures iEHR adoption across the country. This includes digitization of client and provider registries and four clinical components (diagnostic images, laboratory test results, dispensed drugs and clinical reports/immunizations) [4]. Twelve out of the thirteen PTs in Canada have fully digitized registries in place. The clinical components of iEHR are at varying levels of availability across Canada; all PT’s have at least two clinical components available and five PTs have all four clinical components available. About 250,000 health professionals, approximately half of Canada’s anticipated potential physician, nurse, pharmacist, and administrative users, indicate that they electronically access data from outside their main practice setting, such as those found in PT lab or drug information systems. As of January 2015, there were more than 91,000 active iEHR users accessing two or more of the clinical components in a given month [5]. Many more clinicians would access only one clinical component, such as a comprehensive medication profile.

Evaluation of the value of health information exchange is relatively new. A handful of reviews have been published from the US assessing the impact of HIEs. In 2010, a systematic review of the evidence of HIE was published but focused only on primary care. The authors found improvements in referrals and access to test results based on 3 articles but underlined the shortage of empirical evidence to draw conclusions [6]. Subsequently in 2011, a review on the impact of HIE on healthcare outcomes found 5 relevant studies that mostly focused on health care utilization. The authors did not find conclusive evidence on HIEs given the early stages of HIE operation and the paucity of well-designed published research [7]. In the following 3 years, there has been an increase in the published literature but limitations on the generalizability of the resulting evidence remain. A systematic review in 2014 found 85 relevant papers that addressed a mix of health outcomes, efficiency, utilization, costs, satisfaction, usage, sustainability, and/or attitudes and barriers related to HIEs [8]. Most focused on specific care settings and a few HIEs. This limited the generalizability of findings. The review reported low-quality evidence of reduced emergency department use or costs, but effects elsewhere were uncertain. The authors also indicated that “all stakeholders claim to value HIE, but many barriers to acceptance and sustainability exist.” Another review published in 2015 focused on a narrower set of outcomes (costs, use, and quality). It identified 27 studies and came to similar conclusions, highlighting the lack of high quality studies comparable across settings to provide generalizable benefits of HIE [9]. The most recent systematic review of HIE to come out of the US, builds on the evidence from earlier studies identifying 34 studies reporting clinical, economic, population outcomes as well as patient and clinician perceptions of outcomes. The authors concluded there was low-quality evidence to support the value of HIE in reducing duplicate testing, emergency department costs, hospital admissions and improving public health reporting, ambulatory quality of care and disability claims processing. In addition, the review found that clinicians perceptions of the value of HIE were positive. The review also concluded that there was insufficient evidence on the impact of HIE on patient outcomes [10].

In Canada, a number of researchers have examined the iEHR and its components. A recent discussion paper summarizing the benefits of ehealth investments in Canada identified a number of studies that have studied the value of sharing specific types of information (components of iEHR clinical domains) across care settings [11]. For example, evaluations of the benefits of diagnostic imaging information systems (DI) have found that picture archiving communication systems (PACS) provide benefits for health providers such as better access to information, quicker turnaround time, reduced time searching for films, as well as better care, cost savings and productivity improvements for radiologists and technologists particularly in remote, rural locations [12]. Another study focusing on costs of DI found mixed evidence [13]. Similarly, pan-Canadian evaluations of drug information systems (DIS) across the country found benefits such as fewer adverse drug events, improved medication compliance, reduced medication abuse, reduced inappropriate prescription filling, enhanced quality of admission reconciliation practices, and increased productivity for providers [14–17]. DIS studies also found that benefit realization was driven by clinician efforts, sound change management, solution design, interoperability, and accuracy of medication histories [14, 16, 18]. These types of studies provide increasingly rich understanding of the value and success factors related to sharing of particular types of information among authorized clinicians, but the impact of iEHRs as an integrated approach to sharing many different types of clinical data has not been studied as extensively.

This study targets this gap in the literature, with a particular focus on the Canadian experience. It synthesizes iEHR user views from six PTs assessed by surveys. The surveys include user perspectives on system, information, and service quality; iEHR use and user satisfaction; and net quality and productivity benefits. The goal is to improve understanding of how iEHR users perceive the effects of these information sources, with a view to helping advance future use, benefit, and evaluation of iEHRs.

## Methods

**Fig. 1:**
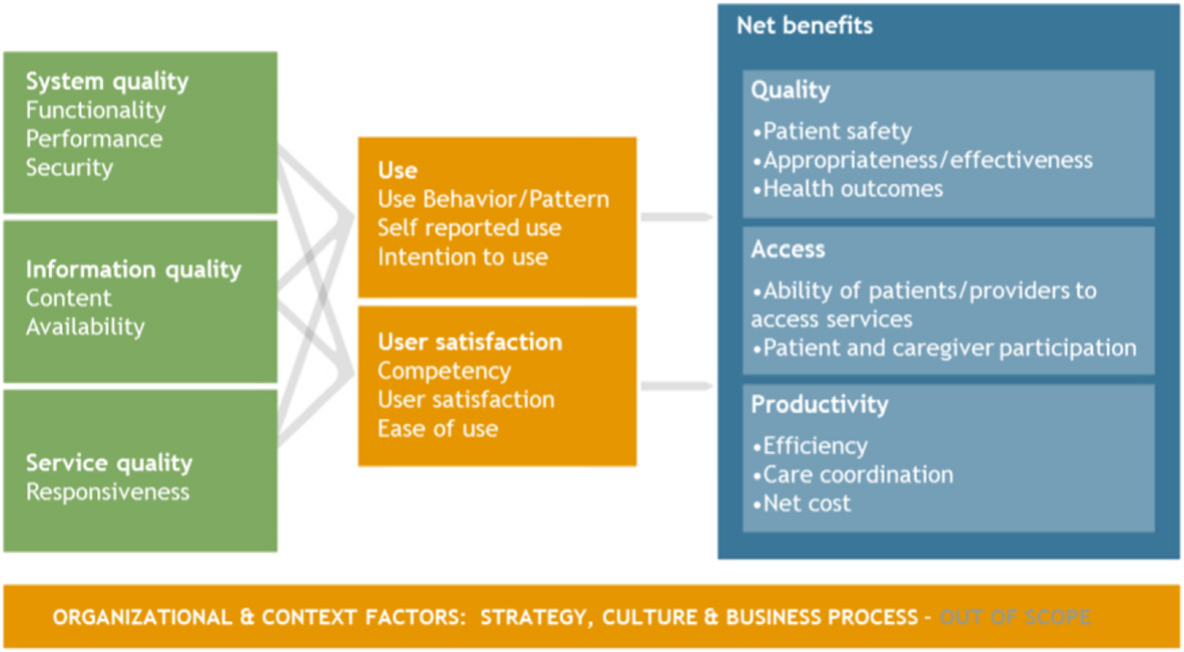
Infoway Benefit Evaluation Framework*

This study synthesizes cross-sectional user surveys conducted as part of provincial/territorial iEHR evaluations. These evaluations were guided by a national Benefits Evaluation Framework (Fig. 1) [19]. This framework draws on work by Delone and McLean regarding evaluating information systems [20]. It describes the relationship between system, information, and service quality; use and user satisfaction; and net quality, access, and productivity benefits. Over time, a series of measurement tools and other resources have been developed based on the framework [21].

### Data sources

One of the measurement tools developed to assist with the evaluation of digital health solutions is a modular System and Use Survey [22]. Designed to be completed by system users, it consists of a core set of questions with Likert-type scales. Each question is aligned with a dimension of the benefit evaluation framework. The survey, and customized versions of it, has been used widely across the country [22].

PT’s with surveys of iEHR users that met the following criteria were included in this study:

- The iEHR was deployed for province/territory-wide use;
- At the time of the survey, the iEHR shared at least two of the core types of clinical data (lab results, medication profiles, diagnostic imaging, clinical reports and immunization history) with authorized users; and
- The System and Use survey (or a customized version of it) had been administered to users either as part of an Infoway project evaluation or by a PT.
- Survey results were available by December 31, 2015.

Table 1 provides details on the six PT’s with iEHR user surveys that met these criteria. In PTs with more than one iEHR System and Use Survey, the most recent data were used. Detailed evaluation results from two of the six PTs have been previously published [23, 24].

**Table 1:** Profile of PT iEHR user survey respondents (at time of evaluation)

	Number of survey respondents (response rate)	Survey distribution method (approximate survey field length)	Professional role of respondents	Clinical settings of respondents	Duration of iEHR use by respondents at time of evaluation	Frequency of iEHR use by respondents	Clinical components of iEHR available at time of survey
1	46 (22 %)	Electronic (n/a)	Nurse, Nurse Practitioner-51 % Physician-15 % Clinical admin-9 % Allied health-9 % Manager, Supervisor-17 %	Acute care, health centres, primary care clinics	Not available	Not available	Laboratory results, Diagnostic imaging, Clinical reports
2	1029 (15 %)	Electronic (1 month)	Nurse, Nurse Practitioner-33 % Physicians-21 % Clerk, medical office assistant-17 % Pharmacist-6 % Allied health-5 % Other-18 %	Acute care, ambulatory, community, emergency, mental health, residential care, palliative, primary care, public health, other	3–5 years-21 % 1–2 years-27 % 7–12 months-16 % 6 months or less-36 %	% of patients in a day for which iEHR is used: 0 % of patients-12 % 25 %–50 % of patients-50 % 75–100 % of patients-22 % Don’t know-17 %	Laboratory results, Diagnostic imaging
3	*n* = 88 (convenience sample, response rate =73 %)	n/a	Nurse-34 % Physician-49 % Clerk, medical office assistant-17 %	Hospital (intensive care unit, Internal medicine, emergency, family medicine)	Not available	Not available	Laboratory results, Diagnostic imaging, Medication profile, Clinical reports
4	244 (N/A)	Electronic (6 weeks)	Nurse, Nurse Practitioner-44 % Physician-12 % Administrative, Support staff-22 % Allied health professional-18 % Other-7 %	Hospital, acute care, primary care, regional or provincial program, other facility, health centre, nursing station, long term care, rehabilitation facility	>24 months-9 % 12–24 months-32 % < 12mos-59 %	Frequency of EHR use in a day: Several times a day-17 % 1–2 times a day-18 % 1–2 times a week-28 % Hardly ever-29 % Never-7 %	Laboratory results, Diagnostic imaging, Medication profile, Clinical reports and Immunization
5	496 (38 %)	Paper (6 weeks)	Nurse-44 % Physician-13 % Clerk, Administrative staff-21 % Other-23 %	Ambulatory, emergency, Private office, inpatient, after hours clinic, other	7–12 months-39 % 6 months or less-61 %	Not available	Laboratory results, Diagnostic imaging, Clinical reports
6	415 (22 %)	Paper (2 months)	Nurse, Physician, and other (breakdown not available)	Ambulatory, emergency, private office, inpatient, after hours clinic, other	>12 months-89 % 7–12 months-7 % 6 months or less-4 %	Not available	Laboratory results, Diagnostic imaging, Medication profile, Clinical reports

While consistent with the same evaluation framework, each survey had specific focus areas. Not all questions from the standard System and Use survey were asked in all cases to reduce respondent burden. Questions to address issues of particular interest to the PT were also added in some cases. Common survey questions across the surveys assessed aspects of system quality (performance, reliability, security); service quality (training and support); overall quality of information (content and availability); user satisfaction and impact on provider productivity and patient quality.

### Data analysis

First, quantitative questions from the six surveys that met the evaluation criteria were compared to identify commonalities. Each survey consisted of anywhere from 21 to 40 questions that were available for study. If a question was asked in two or more surveys, it was retained for further analysis, resulting in a total of 21 questions used in the study. Each of the 21 remaining questions were categorized using the national benefits evaluation framework dimensions. This grouped questions pertaining to system information, or service quality, iEHR use and user satisfaction, or net quality and productivity benefits for analysis.

We then adapted an approach developed by Buntin et al. to classify survey responses as positive, negative, or mixed/neutral [25]. This approach addresses the challenges of aggregating diverse and nuanced findings from a range of studies. It has been previously used in other syntheses of health information technology evaluations [26]. Its application in this study was validated, in advance, by two external subject matter experts.

Specifically, survey responses were classified as follows:

- *Positive:* 50 % or more of survey respondents reported a satisfactory rating or experience in response to a given question, typically representing the top two response options on a five-item Likert-type scale;
- *Negative:* 50 % or more of survey respondents reported a dissatisfactory rating or experience to a given question, typically representing the bottom two response options on a five-item Likert-type scale; and
- *Mixed/Neutral*: Other combinations of responses.

A modified Delphi approach was used to achieve agreement on response classification. One researcher (ST) first conducted this exercise independently. She then met with two other researchers (SH, JZ) on the team to repeat the data classification and analysis exercise, reconciling results to reach consensus.

In addition to this quantitative analysis, qualitative responses to open-ended questions in the System and Use Survey were reviewed. They were used to inform the interpretation of results and discussion.

## Results

Six PTs in Canada had iEHR user surveys that met the inclusion criteria for this study. Together, they had a population of over 11 million in 2014, making up about one-third of the Canadian population [27]. All of the PT iEHRs shared laboratory test results and diagnostic imaging reports from across the jurisdiction with authorized users. Additional information was available in some cases, such as medications, clinical reports and immunization profiles. Users accessed this information in a variety of clinical settings. Acute care, emergency departments, and primary care were among respondents’ most common practice settings at the time of the survey.

The evaluations synthesized in this study took place over an 8 year period, from 2006 to 2014. Together, the surveys had a total sample of 2318 respondents. Sample sizes for individual PTs ranged from 46 to 1027 (see Table 1). The largest groups of respondents were nurses/nurse practitioners, and physicians, ranging from 33 to 51 % and 12 to 49 % respectively. Administrative staff and allied health professionals also accounted for a notable share of respondents in some PTs. Only some surveys asked respondents about how long they had used the iEHR and how often they used it. In most cases, those surveyed had used the iEHR for a year or less, but in one PT, 89 % of respondents reported longer use.

### User perspectives on the iEHR

**Fig. 2:**
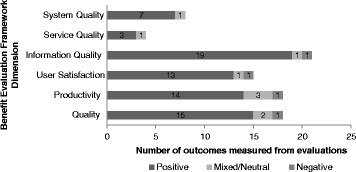
Summary of User Perspectives on iEHR Outcomes

Pooling the evaluations from the iEHRs in the six PTs yielded 21 items that were common to at least two surveys. They assess the following aspects of the benefits evaluation framework: system (2 items), service (1 item), and information (5 items) quality; user satisfaction (3 items); and net productivity (5 items) and quality (5 items) benefits. Table 2 shows a detailed analysis of results in terms of positive, mixed/neutral, and negative outcomes for sub-dimensions of the framework. Figure 2 summarizes results by framework dimension.

**Table 2:** Classification of outcomes measured by iEHR evaluations

Dimension of benefit evaluation framework	Item(s) for comparison across evaluations	Outcomes from evaluations			Total number of evaluations available for comparison (maximum of 6)
		Positive	Mixed/Neutral	Negative	
System quality	Performance and reliability	3	1	0	4
	Security and privacy	4	0	0	4
Service quality	Training and support	3	1	0	4
Information quality	Overall quality of information	3	0	0	3
	Enables access to information previously accessed through another process	3	0	0	3
	Layout and format	3	0	1	4
	Accuracy	4	1	0	5
	Provided quickly/available when needed	6	0	0	6
User satisfaction	Overall satisfaction	4	1	0	5
	Ease of use	4	0	0	4
	Integrated into workflow/makes job easier	5	0	1	6
Productivity	Overall productivity	3	0	1	4
	Efficiency in accessing diagnostic imaging	4	0	0	4
	Appropriate resource utilization (duplication in lab/DI)	3	1	0	4
	Efficiency in accessing lab results	3	0	0	3
	Reduced need to obtain information manually	1	2	0	3
Quality	Clinical decision support	3	2	0	5
	Quality of care	3	0	1	4
	Sharing of information among providers	4	0	0	4
	Information source on patient care provided by another provider/setting	3	0	0	3
	Enhanced ability to coordinate care	2	0	0	2

### System & service quality

In the benefits evaluation framework, the system quality dimension looks at the performance, reliability, and security of iEHR systems. The service quality aspect looks at training and support for use of iEHR systems.

Four surveys assessed aspects of iEHRs system and service quality. In all cases, half or more respondents gave positive rankings to security and privacy. These results were classified as “positive” for the purposes of this analysis. When it came to performance and reliability of the iEHRs, there were three evaluations with positive outcomes and one with mixed/neutral outcomes. Similar results were found for training and support received by iEHR users.

### Information quality

All six evaluations asked users about the quality of information in the iEHR, including its content and availability. The most common question asked was whether the iEHR provided information quickly, sometimes also worded as information being available when needed. All surveys reported positive results in this regard. Likewise, there were consistently positive outcomes in the three surveys that asked about the overall quality of information and the three that asked whether the iEHR enables access to information previously available through another process. When it came to the layout and format of information in the iEHRs, three evaluations had positive outcomes but one had negative outcomes. Likewise, five evaluations looked at the accuracy of information available from iEHRs. Four had positive outcomes and one had mixed/neutral outcomes.

### User satisfaction

All surveys asked about at least some aspects of user satisfaction. The four evaluations that assessed ease of use reported positive outcomes. Overall user satisfaction was measured in five evaluations. Of those, four had positive outcomes and one had mixed/neutral outcomes. Similarly, the integration of an iEHR into workflow and its ability to make the user’s job easier was measured in six evaluations. All but one (which was negative) had positive results.

### Productivity

Respondents in four surveys were asked about the impact of the iEHR on their productivity or efficiency. Overall, three PTs reported positive outcomes and one had negative outcome Only positive outcomes were found in the four evaluations that looked at the iEHR’s impact on efficiency in accessing diagnostic imaging. This parallels findings of significant productivity gains related to digital imaging found in evaluations focused specifically on this question [12]. Similarly, the three evaluations that asked about efficiency in accessing lab results also had positive outcomes. In terms of reductions in duplication of lab tests and diagnostic imaging, three surveys had positive outcomes and one had a mixed/neutral outcome. However, in the three evaluations that asked whether the iEHR reduced the need to obtain information manually, there was only one positive outcome and two mixed/neutral outcomes. This may reflect the fact that many nurses, pharmacists, and physicians continue to report working in hybrid electronic and paper environments on national surveys [28–30].

### Quality

Electronic sharing of patient information is often motivated by the potential to improve the quality of care and patient safety. Users in four PTs were asked whether iEHRs did so overall. Three had positive outcomes in terms of overall quality of care; one had a negative outcome. That said, outcomes from all surveys on the impact on sharing of information among providers (*n* = 4), the iEHR as a source of information on patient care provided by another provider/setting (*n* = 3), and on coordination of care (*n* = 2) reported positive outcomes. Survey results split in terms of clinical decision support. Three of five evaluations showed positive outcomes, with mixed/neutral outcomes in the remainder.

### Summary of user perspectives on iEHR outcomes

In summary, information quality was the most studied area among the evaluations of iEHRs. It had a high ratio of overall positive to mixed/neutral and negative outcomes (19:1:2 respectively). In other evaluative dimensions, there were also more positive than mixed/neutral or negative outcomes. The ratios for user satisfaction, productivity, and quality of care were 13:1:1, 14:3:1, and 15:2:1 respectively.

## Discussion

In Canada, as elsewhere, there is general agreement that health care providers caring for a patient should share relevant patient clinical information electronically in a privacy-sensitive way, regardless of where the patient obtained care [31, 32]. The potential value of doing so is also well understood. The country’s provincial and territorial Health Ministers, for example, issued a joint statement in 2014 declaring that iEHRs are “one of the most transformational innovations in health care in a generation” [33].

This study presents the first cross-jurisdictional assessment of the value of iEHRs from the perspective of their users. This provides unique insights based on the lived experience of those who use these types of systems to access shared patient information, often on a daily basis. By synthesizing survey results from different care settings and jurisdictions, based on different solutions introduced at different times, and that reside within different policy and clinical contexts, the study provides a broad perspective on experience with iEHRs. This diversity can also, as with most research syntheses, present challenges. While there was consistency in the evaluation framework used, surveys were customized to local needs and sample sizes and response rates varied. In addition, this analysis gives equal weight to all studies. This was done intentionally given the wide variation in settings, components of iEHR systems in individual PTs, duration of iEHR system availability, and time of survey. We relied on the actual results from the individual evaluations to classify results as positive, negative or mixed/neutral, rather than attempting to re-interpret them. Any weighting would have been subjective. Instead, we have reflected on some key potential drivers of results below.

One of the study’s limitations is the response rates, which range from 15 to 73 % among the six PT surveys. These surveys were conducted as part of individual PT iEHR evaluation and as such they were all rapid-response surveys with short field times ranging from 1 to 2 months. The relatively low response rates in some evaluations do indicate the need for caution in interpreting the results. Clinician surveys are important in assessing knowledge, attitudes, practice and informing health services and policy research; however, the decline of response rates have been noted as a concern by other researchers [34–36] and echo our experience in this research. Others have found efforts such as reminders, method of survey administration, as well as monetary and non-monetary incentives that might improve declining response rates [34, 36]. Given the rapid nature of the surveys included in this study, such strategies were not utilized to full potential in this research and are a consideration for future evaluations.

There are also other limitations to a study of this type. For example, the included evaluations summarized in this study span an 8 year period from 2006 to 2014. They are not necessarily reflective of the current status of the PT iEHRs, all of which have progressed over time in scope, adoption, and maturity of use. There are also other PT iEHRs in Canada where we did not have similar survey data for comparison. The findings of the study may or may not reflect their users’ views. Furthermore, while the majority of respondents to this survey, like the majority of iEHR users in 2015, access the iEHR through a viewer, the context does differ across provinces, settings and solutions. The association between these factors and the user perception of the system quality and net benefits thus have not been examined in this paper.

In this context, the degree of consistency in the findings across surveys is noteworthy. Overall, users in the six PTs included in this analysis tend to view iEHRs positively. This is true for both the systems themselves and for their effects on productivity and quality of care. For instance, most respondents in all six evaluations agreed that information from iEHRs is provided quickly and is available when needed. The four surveys that asked about information to be shared among care providers—key for coordination of care, particularly for those with chronic conditions cared for by multiple clinicians—also reported positive results. Users also tended to affirm the value of iEHRs in terms of improving productivity and the quality of care.

While results overall were positive, our analysis suggests that system and service quality are a foundation for user satisfaction and net productivity and quality benefits. For example, one PT that had mixed/neutral outcomes on the dependability of their iEHRs had negative findings for overall satisfaction, the ability of iEHRs to make users’ jobs easier, and the impact of iEHRs on productivity and quality of care. Another PT whose users reported that their iEHR system was not well integrated to their workflow also gave poorer ratings for technical support, training, and impact on quality of care. In contrast, one PT stood out for having satisfaction levels far above our “positive” cut off of 50 %. On most dimensions included in the survey, nearly 80 % or more of users rated their iEHR highly, even though they had been using the system for a year or less. This PT had had a coordinated and cohesive approach to iEHR implementation, reflected in user ratings for training and support that met their needs.

This study suggests many directions for future evaluations of health information exchange. Early understanding of the components of the iEHR described a maturity model to benefit realization dependent on the breadth of adoption and richness of the solution [37]. Most of the users surveyed in this study had only been using iEHRs for a short time. Also users’ access to clinical information varied. In some PTs clinicians had electronic access to multiple clinical components such as medication, diagnostic imaging, lab results, and clinical reports all at once. In other PTs, these individual pieces of information were added over time to iEHRs. Studies on sharing particular types of information and specific clinical settings suggest that benefits tend to grow over time, as adoption grows and users gain experience and functionalities or improvements in systems and workflows adapt to user needs [15, 38, 39]. Future studies could confirm if this applies in more complex information exchange environments and identify key enablers and barriers to progress. Future studies will also need to look at the impact of iEHRs for different types of clinician users. Likewise, it would be helpful to be able to compare experiences in Canada and elsewhere to identify best practices and opportunities for mutual learning on a broader scale.

In addition, complementary work to evaluate the effects of information exchange from perspectives other than user views is important. Usability assessment, workflow analysis, and time-and-motion studies are just a few of the approaches that can help to enrich understanding further. In addition, as identified by previous reviews on HIE value, robust study designs are needed to quantify outcomes such as the effect of such solutions on time required to obtain needed patient information; the extent of duplicate testing; changes in hospital readmissions; and improvements in access to care [7–9]. Some of these questions have been explored for particular care settings or types of information. For example, a study in ambulatory care clinics found that settings with more outside connectivity were less likely to report wasted physician and patient time, re-ordering of diagnostic images and lab tests, and being forced to proceed with incomplete patient information [19]. There is potential to build on these types of studies to enrich understanding of the effects of iEHRs and other approaches to health information exchange.

## Conclusion

This study is a first step at providing a pan-Canadian view of user perceptions the effects of using iEHRs (solutions that offer authorized clinicians two or more types of clinical information from across a province/territory) and health information exchange more broadly. Users of iEHRs that provide secure access to patient information collated from across the health system tend to report positive outcomes, including quality of care and productivity.

We hope that its insights will inform future health information exchange efforts and that it will foster continued interest in complementary approaches to evaluation that offer complementary insights on how best to accelerate value through iEHR use.

*Reproduced with permission.

## Abbreviations

DI, diagnostic imaging; DIS, drug information system(s); HIE, health information exchange; iEHR, interoperable electronic health record(s); PACS, Picture Archiving Communication Systems; PT, Province (s) and Territory (ies)
